# Development and Validation of a Smartphone Application for Neonatal Jaundice Screening

**DOI:** 10.1001/jamanetworkopen.2024.50260

**Published:** 2024-12-11

**Authors:** Alvin Jia Hao Ngeow, Aminath Shiwaza Moosa, Mary Grace Tan, Lin Zou, Millie Ming Rong Goh, Gek Hsiang Lim, Vina Tagamolila, Imelda Ereno, Jared Ryan Durnford, Samson Kei Him Cheung, Nicholas Wei Jie Hong, Ser Yee Soh, Yih Yann Tay, Zi Ying Chang, Ruiheng Ong, Li Ping Marianne Tsang, Benny K. L. Yip, Kuok Wei Chia, Kelvin Yap, Ming Hwee Lim, Andy Wee An Ta, Han Leong Goh, Cheo Lian Yeo, Daisy Kwai Lin Chan, Ngiap Chuan Tan

**Affiliations:** 1 Department of Neonatal and Developmental Medicine, Singapore General Hospital, Singapore; 2 SingHealth Polyclinics, Singapore; 3 Nursing Division, Singapore General Hospital, Singapore; 4 Synapxe (formerly Integrated Health Information Systems, IHiS), Singapore; 5 Health Services Research Unit, Singapore General Hospital, Singapore; 6 Yong Loo Lin School of Medicine, National University of Singapore, Singapore; 7 Paediatrics Academic Clinical Programme, Duke-NUS Medical School, Singapore; 8 Lee Kong Chian School of Medicine, Nanyang Technological University, Singapore; 9 Family Medicine Academic Clinical Programme, Duke-NUS Medical School, Singapore; 10 Department of Future Health System, Singapore General Hospital, Singapore; 11 Axrail Private Limited, Singapore; 12 Department of Clinical Pathology, Singapore General Hospital, Singapore

## Abstract

**Importance:**

This diagnostic study describes the merger of domain knowledge (Kramer principle of dermal advancement of icterus) with current machine learning (ML) techniques to create a novel tool for screening of neonatal jaundice (NNJ), which affects 60% of term and 80% of preterm infants.

**Objective:**

This study aimed to develop and validate a smartphone-based ML app to predict bilirubin (SpB) levels in multiethnic neonates using skin color analysis.

**Design, Setting, and Participants:**

This diagnostic study was conducted between June 2022 and June 2024 at a tertiary hospital and 4 primary-care clinics in Singapore with a consecutive sample of neonates born at 35 or more weeks’ gestation and within 21 days of birth.

**Exposure:**

The smartphone-based ML app captured skin images via the central aperture of a standardized color calibration sticker card from multiple regions of interest arranged in a cephalocaudal fashion, following the Kramer principle of dermal advancement of icterus. The ML model underwent iterative development and *k*-folds cross-validation, with performance assessed based on root mean squared error, Pearson correlation, and agreement with total serum bilirubin (TSB). The final ML model underwent temporal validation.

**Main Outcomes and Measures:**

Linear correlation and statistical agreement between paired SpB and TSB; sensitivity and specificity for detection of TSB equal to or greater than 17mg/dL with SpB equal to or greater than 13 mg/dL were assessed.

**Results:**

The smartphone-based ML app was validated on 546 neonates (median [IQR] gestational age, 38.0 [35.0-41.0] weeks; 286 [52.4%] male; 315 [57.7%] Chinese, 35 [6.4%] Indian, 169 [31.0%] Malay, and 27 [4.9%] other ethnicities). Iterative development and cross-validation was performed on 352 neonates. The final ML model (ensembled gradient boosted trees) incorporated yellowness indicators from the forehead, sternum, and abdomen. Temporal validation on 194 neonates yielded a Pearson *r* of 0.84 (95% CI, 0.79-0.88; *P* < .001), 82% of data pairs within clinically acceptable limits of 3 mg/dL, sensitivity of 100%, specificity of 70%, positive predictive value of 10%, negative predictive value of 100%, positive likelihood ratio of 3.3, negative likelihood ratio of 0, and area under the receiver operating characteristic curve of 0.89 (95% CI, 0.82-0.96).

**Conclusions and Relevance:**

In this diagnostic study of a new smartphone-based ML app, there was good correlation and statistical agreement with TSB with sensitivity of 100%. The screening tool has the potential to be an NNJ screening tool, with treatment decisions based on TSB (reference standard). Further prospective studies are needed to establish the generalizability and cost-effectiveness of the screening tool in the clinical setting.

## Key Points

**Question:**

Can the merging of domain knowledge (Kramer principle of dermal advancement of icterus) with current machine learning (ML) techniques create a unique smartphone-based screening tool for neonatal hyperbilirubinemia?

**Findings:**

In this diagnostic study of 546 neonates, the smartphone-based ML application, which used an ML model that incorporated yellowness indicators from the forehead, sternum, and abdomen, underwent internal-external validation against total serum bilirubin (TSB). Pearson *r* was 0.84, sensitivity was 100%, specificity was 70%, and area under the receiver operating characteristic curve was 0.89.

**Meaning:**

These findings suggest the screening tool has good correlation and statistical agreement with TSB, as well as excellent sensitivity.

## Teaser

This diagnostic study evaluates the ability of a smartphone-based machine learning app to predict bilirubin levels in multiethnic Asian neonates using skin color analysis.

## Introduction

Neonatal jaundice (NNJ) affects 60% of term and 80% of preterm infants,^1,2,3,4,5,6,7^ with early detection critical to prevent bilirubin encephalopathy. The criterion standard diagnostic method, total serum bilirubin (TSB) measurement,^8^ is invasive and costly. Noninvasive methods like inspection^7,9,10^ and icterometry^11,12,13,14^ have shown variable accuracy.

Transcutaneous bilirubinometry (TcB) is a rapid, noninvasive tool for screening bilirubin levels in health care settings, using optical spectroscopy to measure bilirubin through the skin.^15,16^ A Cochrane review^17^ of 23 studies with 5058 participants indicated that TcB was generally effective, with varying accuracy due to different study conditions. In Singapore, TcB is the standard method for screening neonates with NNJ in hospitals and outpatient settings. However, its use is limited to health care facilities, requiring calibration, maintenance, and trained personnel. This can lead to increased health care costs, as well as travel expenses and inconvenience for families.

Recently, smartphone applications (apps) have emerged as alternatives for estimating bilirubin levels using digital images of the skin. However, existing apps like Biliscan^18,19,20,21^ have had inconsistent performance across different regions. To the authors’ knowledge, no existing smartphone-based NNJ apps^19,20,21,22,23,24,25,26,27,28^ concurrently use digital images from multiple regions of interest arranged in a cephalocaudal fashion, specifically the forehead, sternum, and abdomen, as predictors for a single bilirubin estimate, which is grounded in the Kramer principle^29^ of cephalocaudal advancement of dermal icterus that underpins bedside NNJ evaluation.

The study team aimed to develop and validate a new artificial intelligence (AI)–based smartphone app for estimating bilirubin levels in a multiethnic neonatal population using skin and/or scleral color images. The study also assessed the association between skin tone, as classified by the Fitzpatrick^30^ skin phototype, and the accuracy of the app. The study team hypothesized that concurrently incorporating skin yellowness from multiple regions of interest, specifically the forehead, sternum, and abdomen, as predictors into the app’s machine learning (ML) model and acquiring a training dataset from a multiethnic neonatal population would enhance its performance.

## Methods

This diagnostic study adhered to the Standards for Reporting of Diagnostic Accuracy (STARD) reporting guideline^31^ and the Transparent Reporting of a Multivariable Prediction Model for Individual Prognosis or Diagnosis (TRIPOD)+AI^32^ updated guidance for reporting clinical prediction models. A dual-phase prospective study from June 2022 to June 2024 was conducted to develop and validate the BiliSG app. We recruited a consecutive sample that included term and late preterm Asian neonates born at 35 or more weeks’ gestation aged within 21 days who were clinically stable regardless of ethnicity. Neonates were excluded if they had skin lesions that interfered with image acquisition or were undergoing phototherapy. Ethical approval for the study was granted by the SingHealth institutional review board.

The study took place at Singapore General Hospital (SGH) and 4 SingHealth Polyclinics located at Bedok, Bukit Merah, Punggol, and Sengkang in Singapore. Parents provided informed consent, and data collected included demographic (gestational age, birthweight, sex, electronic health record–reported ethnicity [Chinese, Indian, Malay, and other ethnicities]), and clinical (cephalohematoma, ABO and Rhesus blood group,^33,34^ glucose-6-phosphate dehydrogenase deficiency status, and phototherapy status) information from electronic health records, as well as skin tone assessed using the Fitzpatrick^30^ scale by study members. Type of feeding (breastmilk, formula, or mixed) and qualitative end-user acceptability information were self-reported. ABO incompatibility in our study was defined as group O mothers with non–group O newborns, group A mothers with group B or AB newborns, and group B mothers with group A or AB newborns.

The study was divided into 2 phases: phase 1 (June 2022 to October 2023) focused on app development and initial ML model creation, while phase 2 (November 2023-June 2024) concentrated on validating the model. There was no public involvement in the study design.

### Methods of NNJ Assessment

Eligible neonates underwent several bilirubin measurements or estimations within the same hour using smartphone-predicted bilirubin (SpB), TcB, and TSB methods. TcB was measured with the Dräger JM-105 bilirubinometer (Dräger Medical GmbH),^35^ which was calibrated on a daily basis. Three measurements from the sternal area were taken, and the mean was recorded.

TSB was measured using capillary heel prick samples analyzed on the Unistat analyzer through direct spectrophotometry^36^ in all study centers. Calibration of the Unistat analyzers occurred every 6 months, along with standardized maintenance at SingHealth Polyclinics and SGH. Quality control was conducted twice daily as per manufacturer standards, with trends tracked using Levy-Jennings plots and Westgard rules. All analyzers took part in proficiency testing to ensure measurement accuracy through peer group and accuracy-based surveys. For neonates who underwent multiple SpB-TSB tests, all results contributed to model development and cross-validation, while only the initial measurement was used for temporal validation.

SpB estimates were obtained using the smartphone app on an Apple iPhone 12 model (Apple Inc) by placing a color calibration sticker card with a central aperture on specific areas (forehead, sternum, and abdomen) or near the eye (eFigure 1 in Supplement 1). Forehead, sternal, and abdominal images were selected based on the Kramer principle,^29^ which describes the cephalocaudal progression of dermal icterus with increasing hyperbilirubinemia. Initially, images were taken from zones 1 to 3, with plans to include limb images (zones 4 and 5) in future studies. Scleral imaging was optional as a backup for participants with darker skin tones,^37^ which may affect accuracy. The app captured and analyzed images of these regions of interest (eFigure 1 in Supplement 1). High-quality images were captured in ambient light and screened for artifacts, with those covering over 35% of the regions of interest excluded. A small group of trained study members (A.J.H.N., M.G.T., V.T., I.E., J.R.D., S.K.H.C., N.W.J.H., and S.Y.S.) performed SpB measurements to minimize variability and were blinded to the results. SpB and TSB measurements were taken within an hour of each other. Clinical management was guided by TcB and TSB measurements as per local clinical practice guidelines.^38^

### Development of the Color Calibration Sticker Card

The color calibration sticker card underwent several iterations to create a final version with a central aperture for skin color assessment and surrounding colored squares. It was designed to correct for light intensity and temperature variations and was printed on matte paper to minimize reflections. The sticker design (eFigure 2 in Supplement 1) also reduced shadows that could affect image accuracy. In the app, the card and skin patches were segmented for analysis, enabling precise color adjustments^39^ based on the card’s patches and improving measurement reliability.

### Inclusion of Predictors in the ML Model

The initial selection of predictors for the ML model was informed by clinical knowledge, using yellowness-related features from the forehead, sternum, and abdomen based on the Kramer principle.^29^ Key predictors included various color metrics (yellow channel in the cyan-magenta-yellow-key [CMYK] color model, blue chromaticity, jaundice eye color index,^40^ and the B channel for yellow and blue components from the LAB color space) and the neonate’s age, as TSB levels typically peak around days 3 to 5.^41,42^ Additional predictors included risk factors for jaundice, such as blood group incompatibility, glucose-6-phosphate dehydrogenase deficiency, preterm birth, cephalohematoma, and exclusive breastfeeding,^43^ as well as factors affecting visual estimation accuracy like skin tone and ethnicity.^44^ The final selection of predictors was automated during the training of the gradient boosted trees model, which sequentially built decision trees that learned from each other’s errors, assessing feature importance based on their contribution to reducing the loss function.

### Iterative Development and Internal-External Validation of the ML Model

During the iterative development and validation of the ML model for predicting bilirubin levels, various techniques—including support vector machine, random forest, extreme gradient boosting, light gradient-boosting machine, gradient boosted trees, and extra trees—were used for their ability to handle nonlinear relationships, their robustness against overfitting, and their ability to provide insights into feature importance. The model output was continuous. The model development employed a 5-fold *k*-folds cross-validation method,^45,46^ dividing the dataset into 5 subgroups. Each fold served once as a test set while the others trained the model, ensuring no overlap between training and testing data for an unbiased evaluation. Missing data were addressed by using the median for continuous variables and the mode for categorical ones, with one-hot encoding for nominal and label encoding for ordinal variables. Feature engineering was performed to create variables like preterm and age above 2 weeks. Data were scaled using a standard scaler before model development. Model performance was assessed using metrics such as root mean squared error (RMSE),^47^ Pearson correlation, and Bland-Altman plots. The final model underwent temporal validation on prospectively recruited neonates. The primary outcomes studied were the linear correlation and statistical agreement between paired SpB and TSB measurements.

### Statistical Analysis

Descriptive analysis summarized the demographic and clinical data of neonates. Pearson correlation coefficient was used to assess the linear relationship between SpB values and TSB. A Bland-Altman plot evaluated the agreement between SpB and TSB within clinically acceptable limits of 50 μmol/L (approximately 3 mg/dL), as prior studies have demonstrated differences of between 2 to 3 mg/dL^24,48^ between TcB, the widely accepted method of screening,^43^ and TSB.

Subgroup analysis by skin tone and sensitivity analysis on poor-quality images was conducted to explore accuracy factors. Sensitivity, specificity, likelihood ratios, and predictive values were calculated using a predefined decision rule (SpB ≥13 mg/dL to predict TSB ≥17 mg/dL) for comparison with prior TcB^49,50^ and smartphone app validation studies.^51^

The smartphone-based ML app’s utility was compared with TcB using receiver operator characteristic curves and the area under the curve (AUC) based on the same decision rule of TcB 13 mg/dL or greater to predict TSB 17 mg/dL or greater. All analyses were performed using Python version 3.9.17 (Python Software Foundation), following the prespecified study protocol.

The sample size was estimated based on clinically acceptable limits of agreement for TSB (±3 mg/dL). Using data from Aune et al,^23^ a conservative mean difference of 0.5 mg/dL and a SD of 1.3 mg/dL indicated that 463 paired measurements were needed to detect agreement based on a 95% CI for the limits of agreement at 80% power and maximum allowable difference of 3 mg/dL. Accounting for a 15% loss of information due to image quality or participant withdrawals, 545 babies would need to be recruited, contributing to 545 pairs of measurements for model development (70%) and prospective validation (30%). A 2-sided *P* value less than .001 was considered significant.

## Results

**Figure 1.**
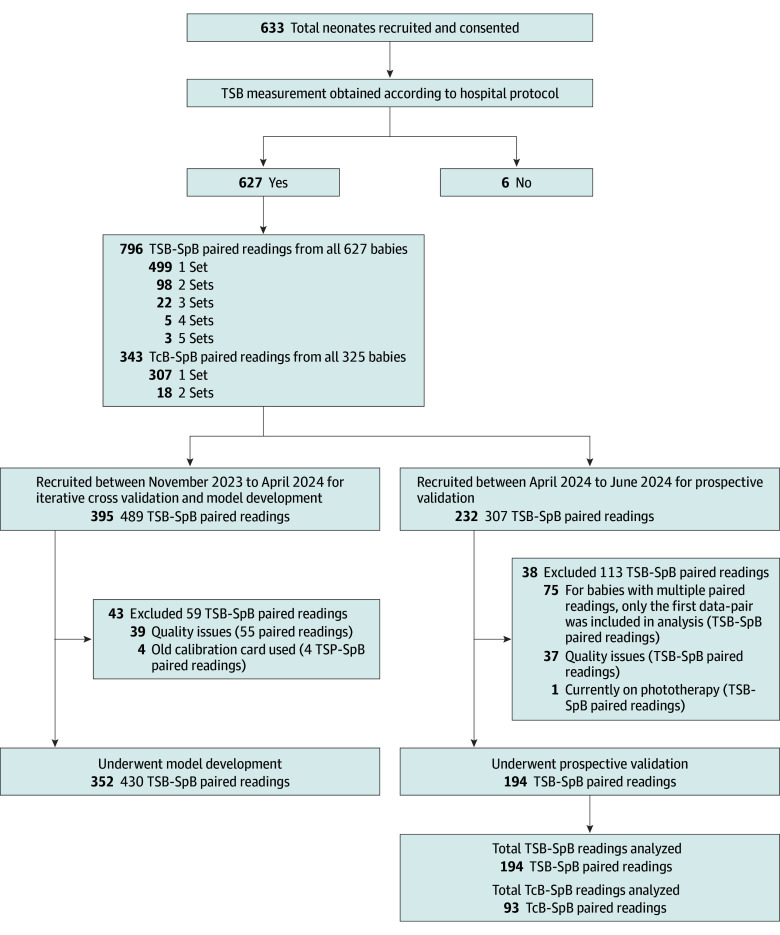
Consort Diagram of Patient Recruitment SpB indicates screening tool bilirubin; TcB, transcutaneous bilirubinometry; TSB, total serum bilirubin.

Between November 2023 and June 2024, 633 unique neonates were recruited at SGH and 4 primary care clinics (Figure 1). A total of 627 neonates had paired TSB and SpB readings for analysis, with 499 (79.6%) having 1 reading, 98 (15.6%) 2 readings, 22 (3.5%) 3 readings, 5 (0.8%) 4 readings, and 3 (0.5%) 5 readings.

During the iterative development and cross-validation phase, 395 neonates were recruited, but 43 were excluded for poor image quality, leaving 352 for cross-validation. After finalizing the ML model in mid-April 2024, 232 additional neonates were recruited, with 37 excluded for image quality and 1 for phototherapy, resulting in 194 for temporal validation.

**Figure 2.**
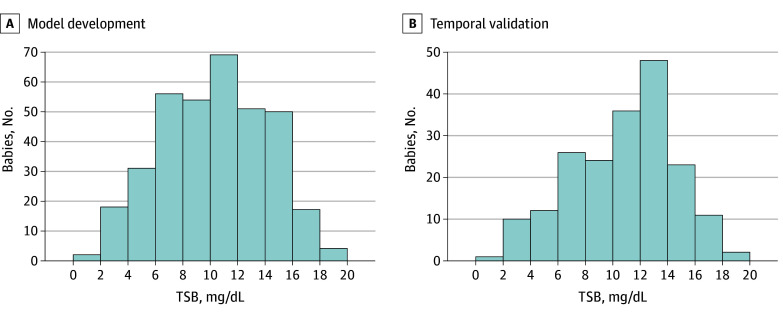
Distribution of Total Serum Bilirubin (TSB) Levels SI conversion factors: To convert bilirubin to μmol/L, multiply values by 17.1.

Demographic data (Table) showed an ethnic distribution similar to the national population (315 [57.7%] Chinese, 35 [6.4%] Indian, 169 [31.0%] Malay, and 27 [4.9%] other ethnicities), with a median (IQR) gestational age of 38.0 (35.0–41.0) weeks and birth weight of 3045 (1975-4514) grams and 285 male neonates (52.4%). Glucose-6-phosphate deficiency was present in 15 neonates (2.7%), and ABO group incompatibility was documented in 149 (27.7%). TSB level distribution (Figure 2) showed comparable patterns between the prospective validation dataset and the training set, particularly for TSB values 10 mg/dL or less and 14 mg/dL or greater, despite a slight leftward skew in the validation dataset.

**Table.** Demographic and Clinical Characteristics

Characteristic	Patients, No. (%)		
	All (N = 546)	In model development (n = 352)	In prospective validation (n = 194)
Gestational age, median (range), wk	38.0 (35.0-41.0)	38.0 (35.0-41.0)	38.0 (35.0-41.0)
Full term	514 (94.1)	326 (92.6)	188 (96.9)
Preterm	32 (5.9)	26 (7.4)	6 (3.1)
Hour of life, median (range), h	120.0 (24.0-504.0)	120.0 (24.0-504.0)	118.5 (24.0-504.0)
Birthweight, median (range), g	3044.5 (1975.0-4514.0)	3046.5 (1975.0-4284.0)	3032.5 (2094.0-4514.0)
Sex			
Female	260 (47.6)	168 (47.7)	92 (47.4)
Male	286 (52.4)	184 (52.3)	102 (52.6)
Ethnicity			
Chinese	315 (57.7)	194 (55.1)	121 (62.4)
Indian	35 (6.4)	22 (6.3)	13 (6.7)
Malay	169 (31.0)	118 (33.5)	51 (26.3)
Others^a^	27 (4.9)	18 (5.1)	9 (4.6)
TSB, mean (SD), mg/dL	10.51 (3.83)	10.34 (3.84)	10.82 (3.80)
Range of TSB, mg/dL	0.88-20.46	1.64-19.94	0.88-20.46
Skin tone type			
I	131 (24.0)	41 (11.6)	90 (46.4)
II	340 (62.3)	241 (68.5)	99 (51.0)
III	68 (12.5)	63 (17.9)	5 (2.6)
IV	7 (1.3)	7 (2.0)	0
Mode of delivery			
Spontaneous	252 (46.2)	151 (42.9)	101 (52.1)
Cesarean	187 (34.2)	112 (31.8)	75 (38.7)
Operative vaginal	107 (19.6)	89 (25.3)	18 (9.3)
Presence of cephalohematoma	11 (2.0)	3 (0.9)	8 (4.1)
ABO incompatibility	149/538 (27.7)	101345 (29.3)	48/193 (24.9)
Glucose-6-phosphate dehydrogenase deficiency	15 (2.7)	11 (3.1)	4 (2.1)
RH incompatibility	4 (0.7)	4 (1.1)	0
Scleral images obtained	10 (1.8)	7 (2.0)	3 (1.5)

Abbreviation: TSB, total serum bilirubin.

SI conversion factor: To convert bilirubin to μmol/L, multiply values by 17.1.

^a^ No subcategories were collected under Other.

### Predictors

In the final ML model, yellowness-related predictors from the forehead, sternal, and abdominal regions were among the top predictors. The discernible yellowness gradient between the sternal and abdominal regions decreased at higher TSB levels, particularly higher than 12 mg/dL (eFigure 3 in Supplement 1), while no consistent gradient was observed between the forehead and other regions. Scleral images were excluded from analysis due to successful capture in only 10 participants.

To improve interpretability of the model, Shapley additive explainability tools (refer to eMethods and eFigure 4 in Supplement 1) were employed, revealing that the neonate’s hour of life was consistently 1 of the top predictors. This aligns with clinical knowledge that TSB levels typically rise in the first few days of life.^41,42^ Other significant features included yellowness-related attributes from skin images, such as the CMYK yellow channel, the LAB B channel, and the jaundice eye color index value, indicating the model’s reliance on the degree of yellowness in the images for predictions.

### Cross-Validation and Temporal Validation of the Final ML Model

**Figure 3.**
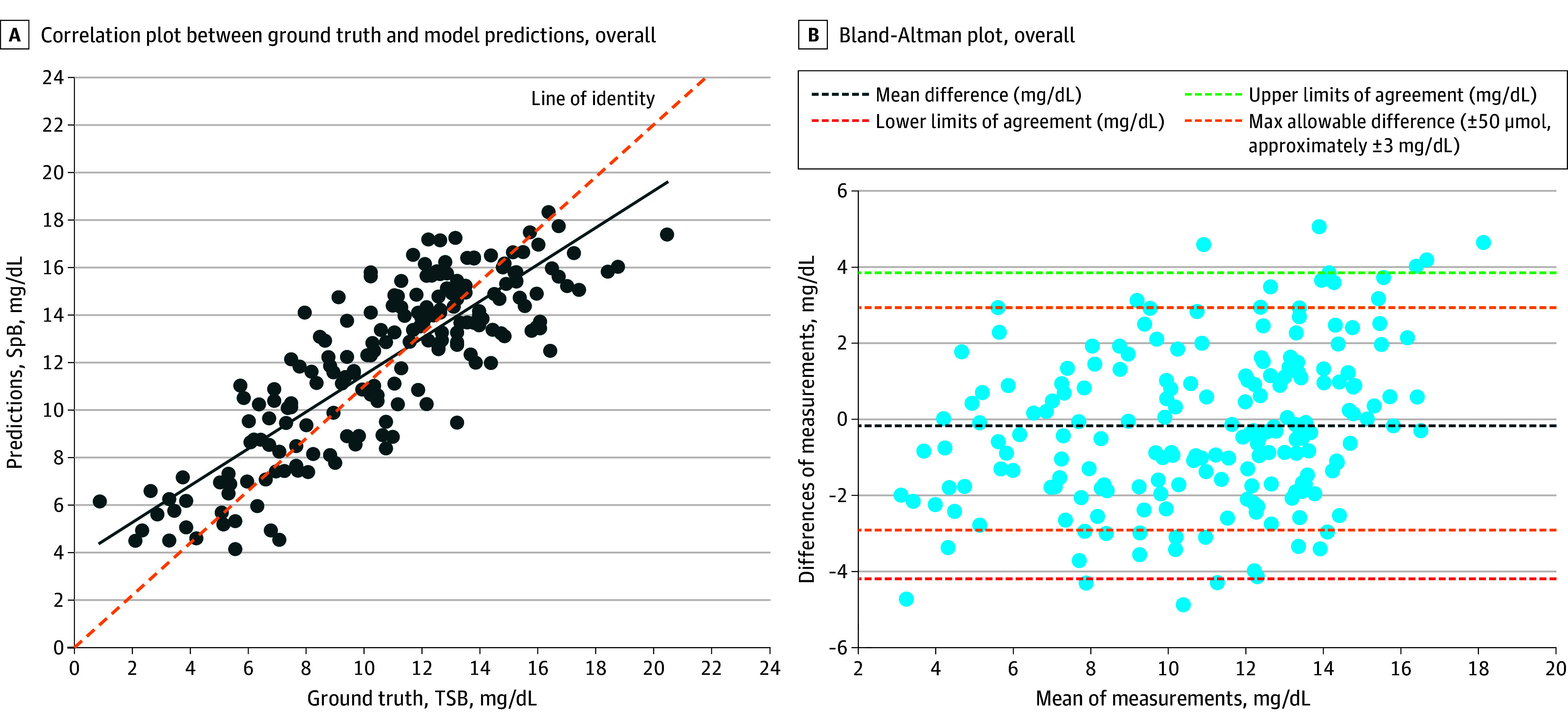
Pearson Correlation and Bland-Altman Plot Between Smartphone-Predicted Bilirubin (SpB) and Total Serum Bilirubin (TSB) (n = 194) A, Each dot represents a paired observation between the reference standard TSB and the SpB for individual measurements. The scatter of the dots shows the relationship between the actual and predicted values. The solid line represents the line of best fit through the data points, showing the trend in the relationship between reference standard TSB and SpB. B, Each dot represents the difference between the SpB and reference standard TSB levels for each observation, plotted against the mean of the SpB and reference standard TSB values for that observation.

The gradient boosted trees model was selected as the final algorithm due to its consistent performance, achieving an RMSE of 2.41 mg/dL and a Pearson correlation of 0.77 (*P* < .001) between SpB and TSB, with 76% of data pairs within a clinically acceptable difference of 50 μmol/L (approximately 3 mg/dL). After finalizing the ML model, 194 individuals were prospectively recruited for temporal validation, which revealed a strong correlation between SpB and TSB (refer to eTable 1 in Supplement 1; Figure 3A), with a Pearson coefficient of 0.84 (95% CI, 0.79-0.88; *P* < .001). For ethnic groups, the coefficients were 0.86 (95% CI, 0.80-0.90; *P* < .001) for Chinese, 0.91 (95% CI, 0.73-0.97; *P* < .001) for Indian, and 0.81 (95% CI, 0.69-0.89; *P* < .001) for Malay neonates. For Fitzpatrick skin phototype II and III, coefficients were 0.85 (95% CI, 0.79-0.90; *P* < .001) and 0.92 (95% CI, 0.20-0.99; *P* = .03), respectively. A sensitivity analysis of 231 babies (194 without artifacts and 37 with significant artifacts affecting >35% of regions of interest) showed a Pearson coefficient of 0.81 (95% CI, 0.76-0.85; *P* < .001).

The Bland-Altman plot (Figure 3B) indicated that 82% of paired measurements were within the maximum acceptable difference, with SpB readings slightly lower than TSB, with a mean difference of −0.18 mg/dL (95% limits of agreement [LoA], −4.20 to 3.84 mg/dL). The mean differences among the largest ethnic groups were similar: −0.21 (95% LoA, −4.01 to 3.59 mg/dL) for Chinese, −0.30 (95% LoA, −3.55 to 2.95 mg/dL) for Indian, and −0.46 (95% LoA, −4.72 to 3.79 mg/dL) for Malay. The final model’s RMSE was 2.06 mg/dL.

### Calibration of ML Model

To assess the calibration of the ML model across the TSB range, the prospective validation set (TSB levels from 0.88 mg/dL to 20.46 mg/dL) was divided into 3 equidistant groups: (1) TSB less than 6.5 mg/dL, (2) TSB between 6.5 mg/dL and 13.0 mg/dL, and (3) TSB greater than 13.0 mg/dL. In each group, 83% (24 of 29), 85% (88 of 104), and 77% (47 of 61) of cases, respectively, fell within the clinically acceptable range of ±3 mg/dL, suggesting an alignment of predictions and underscoring the model’s reliability.

### Diagnostic Accuracy of SpB and TcB Measurements

**Figure 4.**
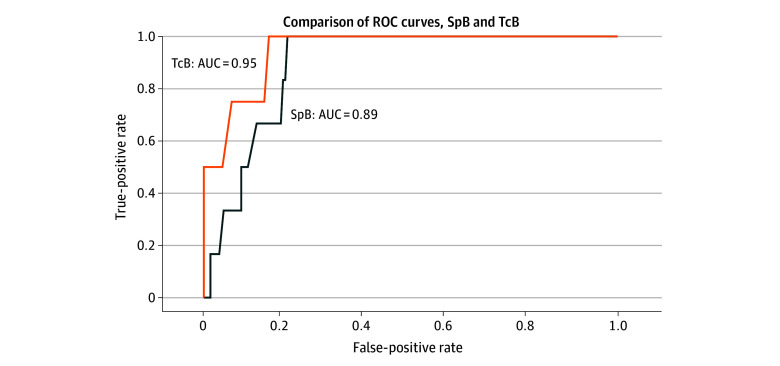
Receiver Operating Characteristic (ROC) Curves of Screening Tool Bilirubin (SpB) and Transcutaneous Bilirubin (TcB) AUC indicates area under the curve.

Using TSB as the criterion standard, the sensitivity and specificity of SpB were 100% (95% CI, 100%-100%) and 70% (95% CI, 63%-76%), respectively, while for TcB, they were 100% (95% CI, 100%-100%) and 51% (95% CI, 40%-61%) (eTable 2 and eTable 3 in Supplement 1). The positive likelihood ratios were 3.30 for SpB and 2.02 for TcB, with both having a negative likelihood ratio of 0.0. The positive predictive value for SpB was 10% (95% CI, 2%-17%), and for TcB, it was 8%(95% CI, 1%-16%), while both had a negative predictive value of 100% (95% CI, 100%-100%). The areas under the receiver operating characteristic curve were 0.89 (95% CI, 0.82-0.96) for SpB and 0.95 (95% CI, 0.87–1.00) for TcB, suggesting diagnostic accuracy for both measurements (Figure 4).

## Discussion

The smartphone-based ML app, developed and validated in the Singapore population, demonstrated a strong correlation (Pearson coefficient of 0.84) between SpB and TSB. It achieved 100% sensitivity and 70% specificity based on the decision rule of SpB 13 mg/dL or greater to predict TSB 17mgl/dL or greater. Its diagnostic accuracy was comparable with TcB.^17^ Screening tool–derived SpB demonstrated a stronger correlation with TSB compared with the pooled correlation coefficient of 0.77 reported by Hedge et al,^52^ which analyzed data from 1733 neonates across 10 studies on smartphone jaundice screening apps that assessed skin yellowness.

The smartphone-based ML app is an innovative NNJ screening tool that has demonstrated strong sensitivity and moderate specificity across a multiethnic population, improving accuracy by using multiple skin regions and color calibration stickers. It can potentially allow remote screening of NNJ by means of home-based SpB measurements and teleconsultations with health care professionals. This approach could reduce the need for frequent clinic visits, especially during epidemics, and has been positively received by parents for its convenience.^58,59^ Future plans involve validating the app’s effectiveness across different smartphone models, different camera specifications, varying lighting conditions, and use by different end-users, as well as exploring various SpB thresholds for guiding the need for TSB assay. A pilot study will assess the app’s safety and acceptability, while a parallel health economics study will evaluate its cost-effectiveness and environmental impact. Ultimately, the goal is to integrate the screening tool with teleconsultation services in primary care clinics in Singapore, establishing a decentralized care model for NNJ screening.

### Strengths and Limitations

The screening tool integrates clinical knowledge, specifically the Kramer principle of cephalocaudal advancement of dermal icterus, with ML by analyzing images from multiple regions, including the forehead, sternum, and abdomen. This approach sets it apart from other smartphone apps that rely on a single region of interest for bilirubin estimation, which can lead to inconsistent results. For instance, using only the sternal area may overlook more severe jaundice that has progressed to the abdomen, affecting the accuracy of predicted bilirubin levels. The study found no consistent yellowness gradient between the forehead and sternal/abdominal regions. This aligns with previous research indicating that bilirubin measurements from the forehead, an exposed area,^53,54^ tend to be underestimated. The authors suggest that natural phototherapy may contribute to this underestimation.

Second, the design of the color calibration sticker card enhanced image quality by eliminating shadows that can affect accuracy of bilirubin estimates. Sensitivity analyses suggested that these shadow artifacts had a significant negative association with correlation, underscoring the importance of this design feature for accuracy.

Third, the large validation cohort of 546 neonates minimized the risk of overfitting and bias, thereby enhancing accuracy.^55^ The app was trained on a diverse dataset which included neonates of different skin tone who were recruited from various ethnic groups in Singapore. The Fitzpatrick phototype scale^30^ provided a more objective classification of skin tone than reliance on ethnic grouping which is often used as a proxy to describe skin tone. This is crucial in a multiethnic country like Singapore.

Fourthly, the study conducted both internal (cross validation-TRIPOD Type 1b) and external temporal validation (TRIPOD Type 2b) of the ML model.^56^ This approach ensured reproducibility and effectiveness of the model in real-world scenarios, providing an objective measure of performance compared with internal validation alone.

The study had several limitations that may affect the generalizability of the smartphone-based ML app. First, only a small number of neonates had Fitzpatrick phototype IV or higher, belonged to minority ethnic groups, or were undergoing phototherapy during recruitment, making it uncertain how well the app applies to these populations without further data from a larger sample.

Additionally, practical challenges in capturing scleral images meant that only a few participants were able to undergo this imaging. Many neonates had their eyelids closed due to drowsiness or sleep, which poses a significant hurdle for NNJ screening apps relying on scleral images, as newborns can sleep for up to 20 hours a day.^57^ Future development may need to consider video-based imaging to address this issue.

The smartphone-based ML app was tested solely on the iPhone 12 due to local cybersecurity policies. Broader validation across various smartphone models, including those running on Android, is necessary to determine its accessibility on different devices.

The study focused on the first 3 zones in the Kramer principle as regions of interest, which are commonly used for TcB measurements.^53^ Zones 4 and 5, which involve limb imaging, were not evaluated due to potential concerns about motion artifacts. Further research is needed to assess whether including these zones could enhance accuracy, particularly for bilirubin levels that approached 20 mg/dL.

While the smartphone-based ML app achieved a sensitivity of 100%, its specificity was only 70%, leading to potential false positives and unnecessary blood draws for TSB testing. This could result in additional costs and emotional stress for parents. Nevertheless, its specificity was still higher than that of TcB, which had a specificity of 51%. As the app is intended to be used as a screening tool, type I errors—misclassifying negative cases as NNJ—are less critical than type II errors, which involve missing actual jaundice cases. Future studies should carefully select SpB thresholds to predict the respective TSB thresholds as per current age-based, risk-stratified local clinical practice guidelines,^38^ emphasizing sensitivity over specificity, to minimize missed cases of severe NNJ.

SpB readings were slightly lower than TSB measurements, with a mean difference of −0.18 mg/dL. This underestimation could lead to missed cases of NNJ that require phototherapy. Further analysis is needed to determine optimal SpB thresholds for the respective TSB thresholds to reduce false negatives. The Bland-Altman plot indicated that while 82% of data pairs fell within a set limit of 3 mg/dL, 9% were over 3 mg/dL lower than the actual TSB. To minimize false negatives, a proposed SpB threshold should be more than 3 mg/dL below TSB levels. For example, using a SpB threshold of 13 mg/dL or greater to predict TSB 17 mg/dL or greater resulted in no false negatives and a sensitivity of 100%.

While 82% of patient data pairs fell within the clinically acceptable limits, 35 cases (18%) exceeded those limits, with half being overestimations and half underestimations. A majority (71%) of these patients had Fitzpatrick skin tone phototype I. The underrepresentation of this skin tone type in the training dataset may have contributed to these discrepancies during temporal validation. Future model refinement will focus on minority skin tones (Types I and IV) and racial groups (Indian and others) to enhance accuracy and inclusivity.

Additionally, although screening was performed by a small group of trained personnel to minimize interoperator variability, operator-specific data were not collected. Future studies will evaluate this variability to bolster confidence in the app’s robustness and reliability in real-world scenarios, particularly for use by parents and caregivers who may be less experienced with such tools. Understanding how the app performs with various operators will help improve its usability and accuracy across diverse settings.

## Conclusions

In this diagnostic study of a new smartphone-based ML app, there was good correlation and statistical agreement with TSB, with sensitivity of 100%. The app has the potential to be an NNJ screening tool, with treatment decisions based on TSB (the reference standard). Further prospective studies are needed to establish the generalizability, and cost-effectiveness of the screening tool in the real-world setting.

## Notes

Supplement 1. **eFigure 1. **Use of BiliSG Application to Acquire Images of Sclera, Forehead, Sternum, and Abdomen **eFigure 2.** Color Sticker Eliminates Shadow Within Central Aperture **eFigure 3. **Sternal-Abdominal Yellowness Gradient Across TSB Range **eMethods.** Selection of Predictors **eFigure 4. **SHAP Analysis of Final Machine Learning Model **eTable 1. **Correlation and Agreement With Total Serum Bilirubin (TSB) for Smartphone-Predicted Bilirubin (SpB) and Transcutaneous Bilirubin (TcB) **eTable 2. **Diagnostic Accuracy of SpB and TcB **eTable 3. **Cross-Tabulation of SpB and TSB Results

Supplement 2.
Nonauthor Collaborators

Supplement 3.
Data Sharing Statement
