# Supplementary material for: Development and Validation of a Smartphone Application for Neonatal Jaundice Screening
Source: JAMA Netw Open. 2024 Dec 11;7(12):e2450260. doi: 10.1001/jamanetworkopen.2024.50260 (PMC11635536; doi:10.1001/jamanetworkopen.2024.50260)
Supplement: Supplement 1. — eFigure 1. Use of BiliSG Application to Acquire Images of Sclera, Forehead, Sternum, and Abdomen eFigure 2. Color Sticker Eliminates Shadow Within Central Aperture eFigure 3. Sternal-Abdominal Yellowness Gradient Across TSB Range eMethods. Selection of Predictors eFigure 4. SHAP Analysis of Final Machine Learning Model eTable 1. Correlation and Agreement With Total Serum Bilirubin (TSB) for Smartphone-Predicted Bilirubin (SpB) and Transcutaneous Bilirubin (TcB) eTable 2. Diagnostic Accuracy of SpB and TcB eTable 3. Cross-Tabulation of SpB and TSB Results [file jamanetwopen-e2450260-s001.pdf]

Supplemental Online Content

Ngeow AJH, Moosa AS, Tan MG, et al. Development and validation of a smartphone application for neonatal jaundice screening. *JAMA Netw Open*. 2024;7(12):e2450260. doi:10.1001/jamanetworkopen.2024.50260

eFigure1. Use of BiliSG Application to Acquire Images of Sclera, Forehead, Sternum and Abdomen
eFigure 2. Color Sticker Eliminates Shadow Within Central Aperture
eFigure 3. Sternal-Abdominal Yellowness Gradient Across TSB Range
eMethod. Selection of Predictors
eFigure 4. SHAP Analysis of Final Machine Learning Model
eTable 1. Correlation and Agreement With Total Serum Bilirubin (TSB) for Smartphone-Predicted Bilirubin (SpB) and Transcutaneous Bilirubin (TcB)
eTable 2. Diagnostic Accuracy of SpB and TcB
eTable 3. Cross-Tabulation of SpB and TSB Results

This supplemental material has been provided by the authors to give readers additional information about their work.

© 2024 Ngeow AJH et al. *JAMA Network Open*.

eFigure 1. Use of BiliSG application to acquire images of sclera, forehead, sternum and abdomen

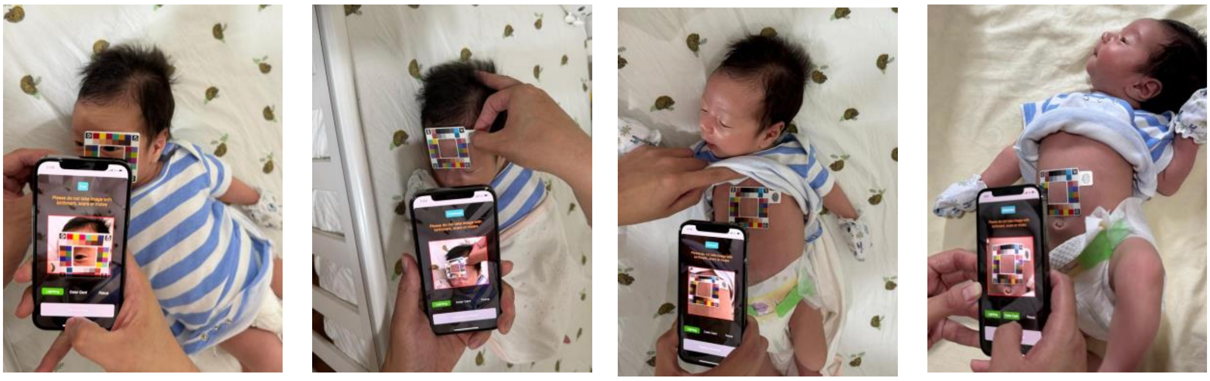

eFigure 2. Color sticker eliminates shadow within central aperture

Colour card (predisposes to shadow due to gap between skin and card)

Colour sticker (eliminates shadow)

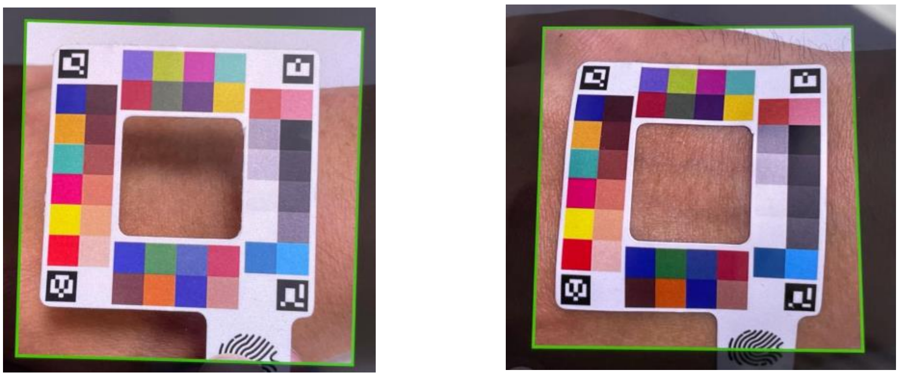

© 2024 Ngeow AJH et al. *JAMA Network Open*.

eFigure 3: Sternal-abdominal ”yellowness” gradient across TSB range

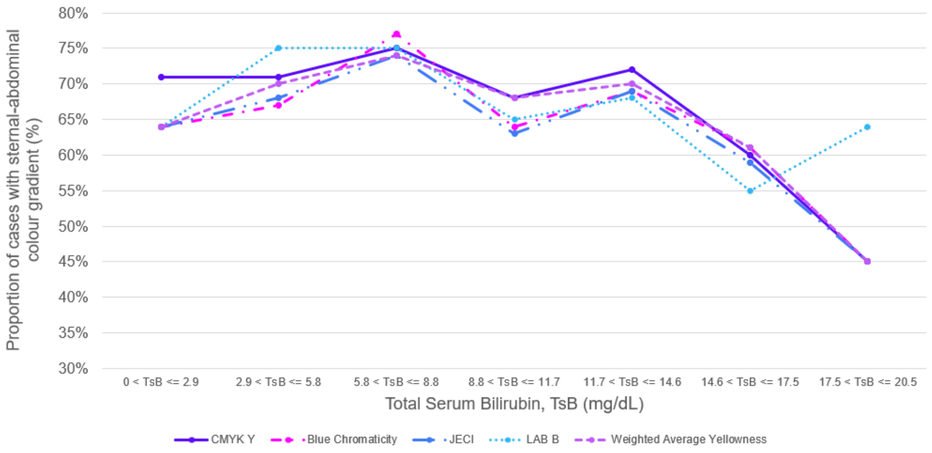

© 2024 Ngeow AJH et al. *JAMA Network Open*.

eMethod. Selection of Predictors

A list of variables was used as predictors in the machine learning model, which is proprietary. The variables include color-related features from various color spaces, such as RGB, LAB, YCrCb, HSV, HSL. Yellowness-related features were computed and included as well.

There were several types of features that were included in the model algorithm:
1. Color-space related features,
2. Yellowness-related features,
3. Clustering features,
4. Gradient features,
5. Similarity features of skin patch and color patches on the calibration card, and
6. User-input features.

Color-space related features referred to features derived from various color spaces such as RGB, LAB, YCrCb, HSV, and HSL.

Yellowness-related features referred to features that specifically picked out the magnitude of yellowness in the skin color. Examples of such features were Y channel of CMYK color space, B channel of LAB color space, blue chromaticity[1] values (which was defined as blue pixel value divided by the summation of the red, green, and blue pixel values. It has an inverse relationship with yellowness), and JECI value.

As for *clustering features*, they were derived in the following manner: For each skin patch, K-Means clustering was done in the RGB color space to get clusters. Cluster centroids' values were extracted and converted to various color spaces. The channels in the respective color spaces which contained yellowness were selected and the channel values were taken. The average of these values (which we call 'yellowness values') of all the centroids was taken to derive the weighted average clustering feature.

Gradient features were used to reflect Kramer Principle[2] of cephalocaudal advancement of dermal icterus with increasing severity of hyperbilirubinemia. The skin patch of all 3 sites was converted into various yellowness-related features and the difference between the skin sites (forehead and sternum, sternum and abdomen) were computed.

Similarity features between the skin patch and color patches on the calibration card were based on the assumption that lighting conditions would impact the entire image equally.

User-input features included gestational age, hour of life, mode of delivery, mother's blood type (Rh/ABO), baby's blood type (Rh/ABO), type of feeding (breastfeeding/formula/mixed), and Glucose-6-Phosphate Dehydrogenase (G6PD) deficiency.

© 2024 Ngeow AJH et al. *JAMA Network Open*.

SHAP analysis was performed to check the feature importance of the predictors in the model. As our final model was an ensemble model, eFigure 4 depicted the SHAP analysis of one of the models in the final ensemble model. eFigure 4. highlighted the most important features used for the model to make its prediction of bilirubin level. As shown, different groups of features emerged as top predictors - yellowness-related features (B value of LAB color space, JECI value, and Y value of CMYK color space), the clustering features (weighted average B channel value from LAB color space and Cb channel value from YCrCb color space), user-input features (hour of life, birth weight), color similarity features between color patches and skin patches, and other features such as color-space related features (S channel value from HLS color space, ratio of green-blue channel in RGB color space).

eFigure 4: SHAP analysis of final Machine Learning model

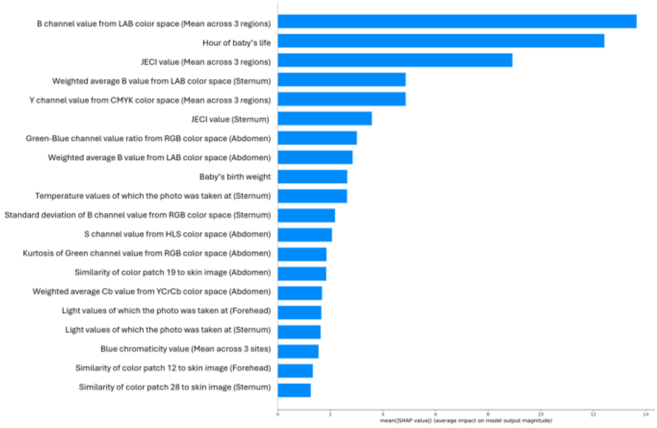

© 2024 Ngeow AJH et al. *JAMA Network Open*.

eTable 1. Correlation and agreement with total serum bilirubin (TSB) for Smartphone-predicted Bilirubin (SpB) and Transcutaneous bilirubin (TcB)					
Measurements	Sample	Pearson correlation coefficient r (95% CI, P-value)	Bland-Altman mean difference (95% LoA, mg/dL)	Min, max difference, mg/dL	Pairs within pre-set limits of 50 μmol/L (approx 3 mg/dL), %
SpB and TSB	Full (n=194)	0.84 (0.79-0.88, P<0.001)	-0.18 (-4.2 to 3.84)	-4.86, 5.08	82
	Chinese (n=121)	0.86 (0.80-0.90, P<0.001)	-0.21 (-4.01 to 3.59)	-3.97, 4.66	82
	Indian (n=13)	0.91 (0.73-0.97, P<0.001)	-0.3 (-3.55-2.95)	-2.74, 2.49	100
	Malay (n=51)	0.81 (0.69-0.89, P<0.001)	-0.46 (-4.72 to 3.79)	-4.86, 4.2	82
	Fitzpatrick Type I (n=90)	0.83 (0.75-0.88, P<0.001)	-0.11 (-4.54 to 4.33)	-4.71, 5.08	72
	Fitzpatrick Type II (n=99)	0.85 (0.79-0.90, P<0.001)	-0.22 (-3.88 to 3.44)	-4.86, 4.2	90
	Fitzpatrick Type III (n=5)	0.92 (0.20-0.99, P=0.027)	-0.8 (-3.14 to 1.54)	-2.43, 0.7	100
TcB and TSB	Full (n=93)	0.90 (0.86-0.93, P<0.001)	-0.73 (-3.43 to 1.98)	-3.62, 2.92	96
	Chinese (n=52)	0.91 (0.85-0.95, P<0.001)	-0.54 (-2.97 to 1.88)	-3.62, 2.1	96
	Indian (n=7)	0.87 (0.33-0.98, P=0.012)	-0.55 (-3.2 to 2.1)	-3.27, 1.46	86
	Malay (n=28)	0.93 (0.86-0.97, P<0.001)	-1.27 (-3.84 to 1.29)	-3.39, 1.93	96
	Fitzpatrick Type I (n=33)	0.86 (0.73-0.93, P<0.01)	-0.35 (-2.9 to 2.21)	-3.27, 2.46	97
	Fitzpatrick Type II (n=55)	0.91 (0.85-0.95, P<0.001)	-0.87 (-3.57to 1.83)	-3.63, 2.92	96
	Fitzpatrick Type III (n=5)	0.92 (0.18-0.99, P=0.028)	-1.67 (-3.88 to 0.53)	-3.27, 0.12	80
SI conversion factors: To convert bilirubin to μmol/L, multiply values by 17.104					

© 2024 Ngeow AJH et al. *JAMA Network Open*.

eTable 2. Diagnostic accuracy of SpB and TcB		
	BiliSG SpB (n=194)<br><br>(No. of positive cases = 6)	TcB (n=93)<br><br>(No. of positive cases = 4)
Sensitivity (95% CI), (%)	100 (100 – 100)	100 (100 – 100)
Specificity (95% CI), (%)	70 (63 - 76)	51 (40 - 61)
Positive likelihood ratio	3.30	2.02
Negative likelihood ratio	0.00	0.00
Positive predictive value (95% CI), (%)	10 (2 - 17)	8 (1 - 16)
Negative predictive value (95% CI), (%)	100 (100 – 100)	100 (100 – 100)
Decision rule: SpB or TcB level ≥13 mg/dL to predict TSB level ≥17mgl/dL		

eTable 3. Cross Tabulation of SpB and TSB results			
		TSB ≥17mg/dL	
		+ (n=6)	- (n=188)
SpB ≥13 mg/dL	+ (n=63)	TP (6)	FP (57)
	- (n=131)	FN (0)	TN (131)

© 2024 Ngeow AJH et al. *JAMA Network Open*.
