# Supplementary material for: Development and Validation of a Smartphone Application for Neonatal Jaundice Screening
Source: JAMA Netw Open. 2024 Dec 11;7(12):e2450260. doi: 10.1001/jamanetworkopen.2024.50260 (PMC11635536; doi:10.1001/jamanetworkopen.2024.50260)
Supplement: Supplement 3. — Data Sharing Statement [file jamanetwopen-e2450260-s003.pdf]

Data Sharing Statement

Ngeow. Development and Validation of a Smartphone Application for Neonatal Jaundice Screening. *JAMA Netw Open*. Published December 11, 2024.
doi:10.1001/jamanetworkopen.2024.50260

Data
Data available: No
